# Acute eosinophilic pneumonia caused by nicotine‐free vaping in an adolescent patient: A case report

**DOI:** 10.1002/rcr2.961

**Published:** 2022-05-13

**Authors:** Yuki Takigawa, Ken Sato, Ayumi Inoue, Momoka Nagae, Tomoyoshi Inoue, Kiriko Onishi, Sho Mitsumune, Hiromi Watanabe, Kenichiro Kudo, Akiko Sato, Keiichi Fujiwara, Takuo Shibayama

**Affiliations:** ^1^ Department of Respiratory Medicine National Hospital Organization Okayama Medical Center Okayama Japan

**Keywords:** acute eosinophilic pneumonia, bronchoalveolar lavage, nicotine‐free vaping, non‐cigarettes, smoking

## Abstract

An 18‐year‐old man was admitted to our hospital with pneumonia 4 days after he initiated vaping. The patient did not show improvement after ceftriaxone and azithromycin treatment. The cell count of the bronchoalveolar lavage fluid (BALF) revealed 64% eosinophils and 18% lymphocytes. Based on the BALF findings, the patient met the current diagnostic criteria and was diagnosed with vaping‐induced acute eosinophilic pneumonia (AEP). AEP caused by nicotine‐free vaping is rare in Japan. Thus, in cases of AEP, the patient's history of cigarette smoking as well as vaping should be considered.

## Graphical abstract

Vaping has become popular among young people and adolescents in Japan because it is easy to obtain and is legally ambiguous. We encountered a case of acute eosinophilic pneumonia caused by nicotine‐free vaping and report our findings to highlight the safety concerns associated with vaping.

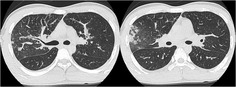

## INTRODUCTION

Acute eosinophilic pneumonia (AEP) is a clinical form of respiratory failure that typically occurs within 1 month of initiating smoking in young adults. Unlike heated cigarettes, nicotine‐free vaping is performed using electronic devices that are free of nicotine and tar, allowing users to perform activities similar to smoking by heating the flavours in a vaping device or electronic cigarette. Vaping has become popular among young people and adolescents in Japan because it is easy to obtain and is legally ambiguous. We encountered a case of AEP caused by nicotine‐free vaping and report our findings to highlight the safety concerns associated with vaping.

## CASE REPORT

The patient was an 18‐year‐old male with no medical history of bronchial asthma. He was not taking any medications, smoking cigarettes or consuming health foods or supplements. In late October 2021 (4 days before admission), he purchased a vape (a vapour‐based electronic cigarette without nicotine and tar) on the Internet and began smoking continuously using the device. Four days later, he developed fever, dyspnoea and anorexia, and was brought to our emergency room. His SARS‐CoV‐2 polymerase chain reaction test results were negative.

**FIGURE 1:**
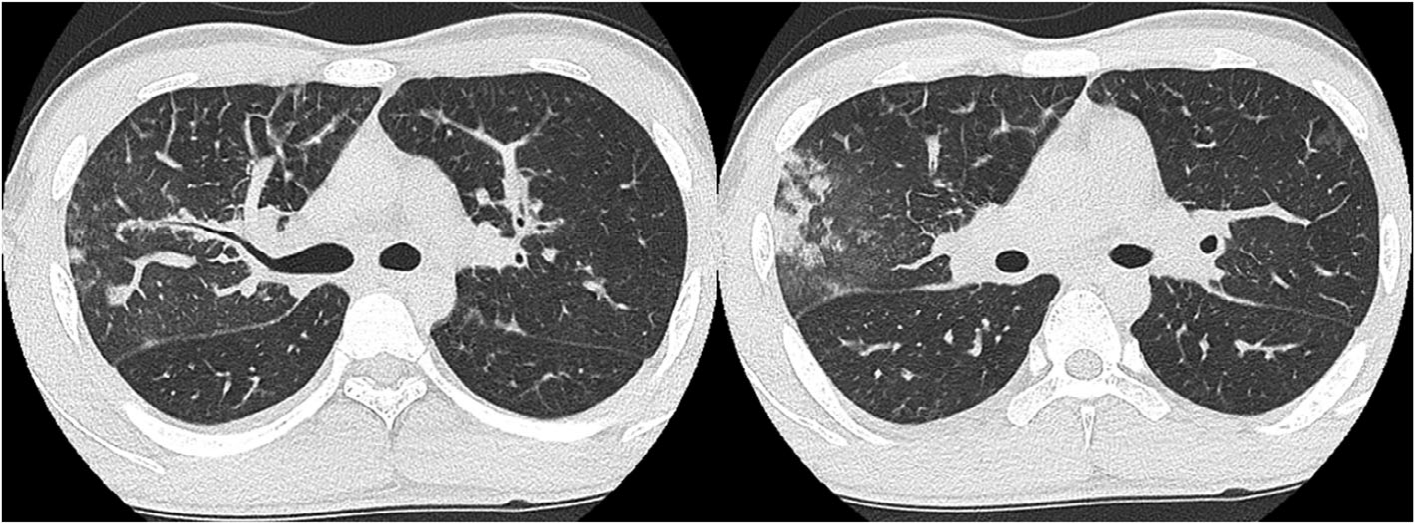
A chest computed tomography scan in the emergency department showed thickening of the bronchovascular bundles and interlobular septal walls in both lungs. In particular, the right upper lobe showed a granular shadow, nodules and ground‐glass opacity under the pleura. A small amount of pleural fluid was observed on the right side.

On admission to our hospital, the patient was conscious, and his vital signs were as follows: temperature, 39.0°C; blood pressure, 114/61 mmHg; heart rate, 138 beats/min; and respiratory rate, 16 breaths/min. The oxygen saturation was 94% on 2 L/min of oxygen inhalation. Physical examination revealed no abnormal findings, except for coarse bilateral crackles in the lungs. A laboratory analysis revealed leucocytosis with predominant neutrophils (white blood cell count 18,370/μl, 90.9% neutrophils and 0.4% eosinophils), and his C‐reactive protein level was elevated (6.28 mg/dl). Krebs von den Lungen‐6, antinuclear antibodies and anti‐neutrophil cytoplasmic antibody levels were all within the normal ranges. A chest radiograph and computed tomography (CT) scan in the emergency department showed thickening of the bronchovascular bundles and interlobular septae in both the lungs. In particular, in the right upper lobe, a granular shadow, nodules and ground‐glass opacity were observed peripherally. A small amount of pleural fluid was observed on the right side. Cardiac enlargement was not observed (Figure 1). Considering the recent history of vaping and CT findings, AEP was considered.

Treatment with ceftriaxone (2 g/day) and azithromycin (500 mg/day) was initiated, but the oxygen status worsened, and the patient required nasal oxygen administration at 4 L/min. Because of the ongoing COVID‐19 pandemic, we were cautious about performing bronchoscopy from the viewpoint of infection control. However, considering the possibility of vapour‐induced AEP based on the findings of various examinations and interviews, bronchoscopic examination was performed on the third day of admission. The cell count of the patient's bronchoalveolar lavage fluid (BALF) from the right B5b bronchi (150 ml of saline was injected and 90 ml was suctioned) revealed 64% eosinophils and 18% lymphocytes, and the BALF was yellow in colour. Based on the BALF findings, the patient met the current diagnostic criteria.^1^ He was diagnosed with vaping‐induced AEP, and prednisone therapy (60 mg) was initiated. The dose was gradually decreased every 3 days. On the fourth day of hospitalization, the patient's fever resolved, and his oxygen status was maintained at room air. The chest radiographic abnormalities had improved.

**FIGURE 2:**
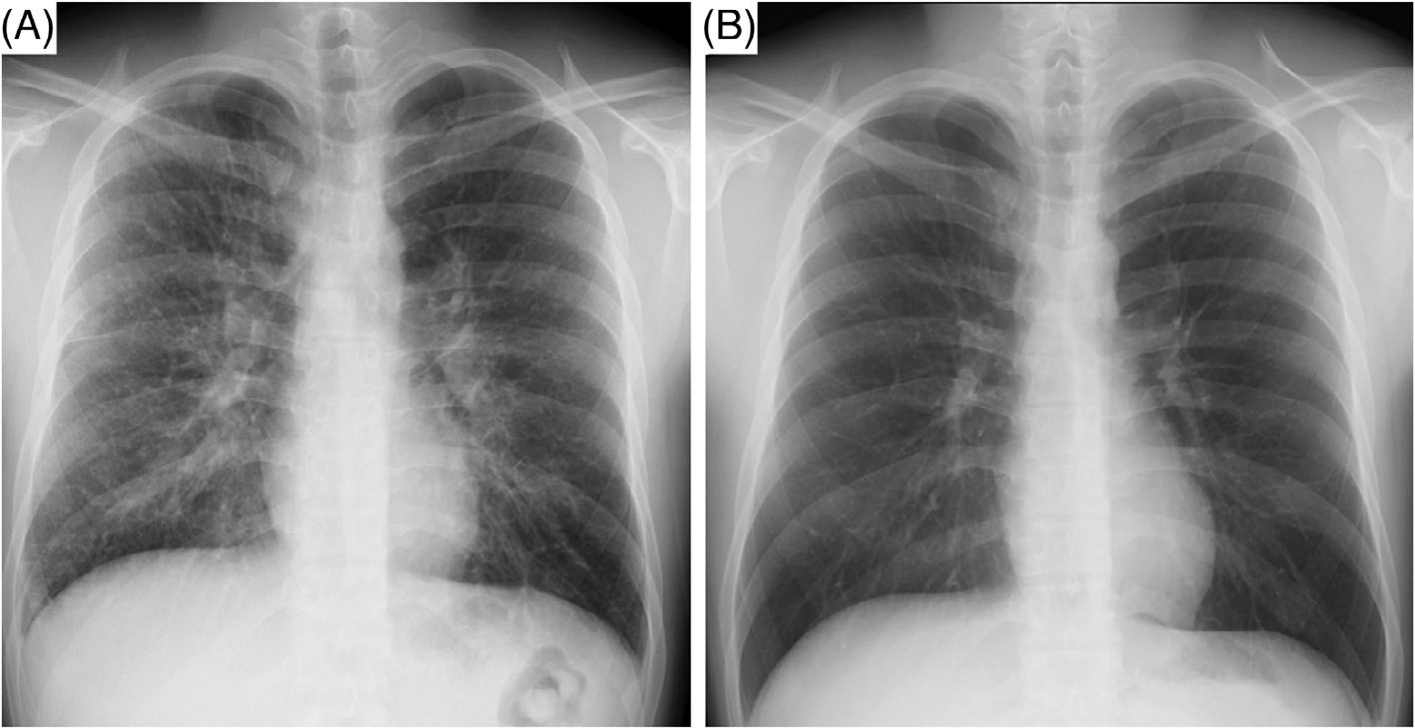
Chest radiograph on admission (A) and discharge (B). The radiograph obtained at discharge shows that the lung abnormalities had disappeared.

Despite the treatment with prednisolone, the blood eosinophil count peaked only on the seventh day (peak white blood cell count 10,980/μl, 21% eosinophils) and steadily decreased thereafter. On the day of discharge from the hospital (12th day of treatment with prednisolone), the eosinophil count improved to normal levels, and chest radiographic abnormalities improved in comparison with those at admission (Figure 2). Respiratory function tests showed significant improvement (Table 1). On the 14th day of steroid therapy, the patient was discontinued prednisolone. At the outpatient visit 1 week after discharge, the absence of AEP recurrence was confirmed. We explained to the patient that re‐smoking would put him at risk of relapse.

**TABLE 1:** Comparison of respiratory function tests

	Before treatment	On the 12th day of prednisolone
VC (L)	1.9	4.52
FEV1.0 (L)	1.56	3.86
PEF (L/min)	2.79	10.1
DL_CO_ (ml/min/mmHg)	12.03	24.53

Abbreviations: DL_CO_, diffusing capacity of carbon monoxide; FEV1.0, forced expiratory volume in 1 s; PEF, peak expiratory flow; VC, vital capacity.

## DISCUSSION

AEP caused by electric cigarettes has been reported in several patients, particularly in the United States.^2^, ^3^ To the best of our knowledge, in Japan, AEP has been reported with nicotine‐containing heated cigarettes^4^ but not with nicotine‐free vaping.

Vape liquids are composed of three main components: polyethylene glycol, vegetable glycerin and flavouring component. Polyethylene glycol is an excipient in numerous medications, healthcare products, cosmetics and foods, and can cause allergic reactions.^5^ In contrast, there are few reports of allergic reactions to glycerin.^6^ The flavouring component differs according to the manufacturer of the product. Although these components use ingredients that are not harmful to the human body, such as those found in beverages and food, the details are unknown. The flavouring component is reported to contain high levels of diacetyl^7^ and is known to be strongly associated with bronchiolitis obliterans syndrome.^8^ As a result, the exact cause of AEP cannot be clearly determined in the present case, even when AEP is shown to be clearly caused by vaping. However, we consider that vaping‐induced AEP might have been caused by polyethylene glycol and/or flavouring components rather than glycerin.

The proportion of adolescents who vaped at least 20 days in a month increased from 20% in 2017 to 28% in 2018.^9^ Therefore, when considering AEP, interviews should include questions about the history of electric or heated cigarette usage in addition to the history of cigarette smoking. In this case, we were unable to obtain a smoking history from the patient himself at first, but a family member (the patient's grandmother) living with the patient told us that he had purchased a vape and started vaping. Leventhal et al. reported an association between electronic cigarette use and initiation of combustible tobacco product smoking in early adolescence. Thus, in patients who smoke e‐cigarettes, we should pay attention to their medical histories to see if they also smoke normal cigarettes.^10^

Notably, in the early stages of AEP, only the neutrophil count may be elevated, and the eosinophil count may not be elevated. In fact, blood eosinophil counts do not increase during the acute phase in the clinical course of AEP in 75% of patients,^11^ but in some cases, eosinophil counts increase during the recovery phase of 2–10 days.^12^ There have been reports of azithromycin‐induced eosinophilic side effects, which cannot be completely ruled out. The findings in the present case appear to be consistent with these results, indicating the need for caution while interpreting the clinical course and laboratory findings.

In this case, the corticosteroid response was very good, and oxygen administration was not necessary from the day after prednisolone administration at rest; therefore, corticosteroids were tapered off and discontinued within a short period. A previous study reported that the duration of corticosteroid treatment could be shortened to 2 weeks even in patients with respiratory failure associated with AEP.^13^

More than 1000 new cases of e‐cigarette, or vaping, product use associated lung injury (EVALI) have been reported in the United States.^14^, ^15^ EVALI was defined as a history of use of e‐cigarette or vaping device within 90 days prior to symptom onset.^14^ The patient must have a history of e‐cigarette‐related product use prior to the onset of respiratory symptoms, and a chest CT scan showing shadows in the bilateral lung fields, without any respiratory infection. Histopathological findings of EVALI showed airway‐centred acute lung injury, often with severe bronchiolitis accompanied by marked mucosal oedema, sloughing of bronchiolar epithelium and peribronchiolar organization.^16^ EVALI should be considered in the differential diagnosis for the patient, but this case met the current diagnosis of AEP and the clinical course, including BALF finding, was compatible with the diagnosis of AEP.

We encountered a case of AEP caused by vaping. We report this case to highlight the safety concerns associated with nicotine‐free vaping and the importance of paying attention to the patient's history of cigarette smoking and vaping in cases of AEP.

## AUTHOR CONTRIBUTION

Yuki Takigawa and Ken Sato wrote the manuscript, which was then reviewed by all co‐authors.

## CONFLICT OF INTEREST

None declared.

## ETHICS STATEMENT

The authors declare that appropriate written informed consent was obtained for the publication of this manuscript and accompanying images.

## Data Availability

The data that support the findings of this study are available from the corresponding author upon reasonable request.
